# The adoption of precision agriculture enabling technologies in Swiss outdoor vegetable production: a Delphi study

**DOI:** 10.1007/s11119-022-09889-0

**Published:** 2022-03-04

**Authors:** Jeanine Ammann, Christina Umstätter, Nadja El Benni

**Affiliations:** 1 Agroscope, Research Division on Competitiveness and System Evaluation, Tänikon 1, 8356 Ettenhausen, Switzerland; 2 Thünen Institute of Agricultural Technology, 38116 Braunschweig, Germany

**Keywords:** Smart farming, Technologies, Drivers, Barriers, Experts, Agriculture

## Abstract

Digital technologies are a promising means to tackle the increasing global challenges (e.g., climate change, water pollution, soil degradation) and revolutionising agricultural production. The current research used a two-stage Delphi study with 34 experts from various domains, including production, advisory and research, to identify the key drivers and barriers, the most promising technologies and possible measures to support technology adoption in Swiss outdoor vegetable production. Combining these experts’ views, the method provides realistic scenarios for future development. In Round 1, open-ended questions were used to collect the experts’ opinions. These were then transformed into closed-ended questions for Round 2, where controlled feedback was provided to the experts. Twenty-six experts participated in both rounds, resulting in an overall response rate that was comparably high (76%). It was found that economic factors were important drivers and barriers in technology adoption and, consequently, the experts recommended financial measures to support this adoption. The practical relevance of new technologies provided through communication and education holds further potential in terms of their promotion. These findings are valuable beyond the research field. Educators and policy makers can build on the results and optimally align their efforts to target technology adoption and contribute to more sustainable agriculture.

## Introduction

The use of digital technologies can help to tackle the increasing challenges in agriculture (Busse et al., 2014; Finger et al., 2019; Walter et al., 2017). These include the growing global demand for food (Hickey et al., 2019); environmental challenges, including climate change, loss of biodiversity, soil degradation, and water pollution (Barrett & Rose, 2020; Rial-Lovera et al., 2017), and the rising social pressure reflected in increasingly strict agricultural policies (Finger et al., 2019). The use of new technologies can help farmers optimise input allocation and thereby contribute to lower costs, increased outputs and higher resource efficiency (Batte & Arnholt, 2003; Shockley et al., 2011). More precisely, the use of sensors can contribute to better monitoring of a farm so that inputs, such as fertilisers or pesticides, can be applied according to its needs (Walter et al., 2017), assuming that the farmer can use the data collected on the farm and put it into practice. Precision agriculture enabling technologies (PAT), such as driver assistance systems and electronic measuring systems (Groher et al., 2020), can also have social impacts, such as the potential increase of wellbeing at work through the reduction of repetitive tasks or driver relief (Holpp et al., 2013), or the potential decrease in working time (Ayerdi Gotor et al., 2019). However, despite this potential of PAT, adoption rates differ widely across geographic regions and between different technologies (Barnes et al., 2019; Lowenberg-DeBoer & Erickson, 2019a). For example, in Swiss outdoor vegetable production, driver assistance systems are commonly used (87% adopters), whereas sensors are less frequently used (31% adopters; Groher et al., 2020).

The possible barriers to PAT adoption are manifold. One obstacle relates to farmer education, which is crucial in providing the skills necessary for technology adoption (Michels et al., 2020; Paustian & Theuvsen, 2016), as the use of PAT often requires specific training. In many countries, this educational provision is still in the early stages of development (e.g., Eastwood et al., 2019; Eastwood et al., 2012). The increasing demand to include this topic in agricultural education can be seen in Switzerland, where a new teaching module on smart farming was introduced in 2020 (Lampart, 2021). Another important barrier to adoption which has been widely acknowledged across the literature relates to the economic costs associated with switching production systems to new digital technologies (Barnes et al., 2019). In a similar vein, previous research found that sufficient capital was the best predictor for adoption (Baumgart-Getz et al., 2012). A final barrier to adoption is the available infrastructure, such as network coverage. For instance, while many devices rely on network access, a significant number of farms in the United Kingdom currently lie outside the range of 4G (Tang et al., 2021). Similar findings apply to most countries, where the current availability of network connection is not sufficient (USDA, 2019).

While the adoption rates for PAT are well documented in certain U.S. states and in Australia, they are not as well explored in Europe (Barnes et al., 2019; Kutter et al., 2009; Paustian & Theuvsen, 2016). In their review, Lowenberg-DeBoer and Erickson (2019b) noted that most precision agriculture adoption studies they cited hypothesised about the improvements needed to accelerate technology adoption. Those hypotheses can be summarised in the following three points: First, and as mentioned above, technology costs need to be reduced. Second, more reliable decision rules are required; farmers want to know when to use which applications and what the results will be. Third, added value needs to be demonstrated and profits made visible (Lowenberg-DeBoer & Erickson, 2019b).

In the current research, the Delphi method was used to explore the current and future development of PAT in Swiss outdoor vegetable farming. The analyses and prognoses obtained through the Delphi process are of crucial importance in both theory (e.g., better understanding of the processes) and practice (e.g., when defining policy measures or supporting farmers to adapt and improve their production systems). Furthermore, given that the views on PAT often differ between stakeholders, with farmers tending to be unsure about the investment in and potential of the technologies and experts tending to expect promising developments (Balafoutis et al., 2020), the use of the Delphi method made it possible to bring these different views together in a combined and realistic prognosis.

Previous research (Groher et al., 2020) analysed the current levels of adoption in Swiss agriculture. The present study takes the next step by adding to this existing evidence. It identifies possible future scenarios and estimates the future rates and development of adoption. The Delphi method is an established tool for scientific forecasting, the main aim of which is to obtain high-quality responses from a selected panel of experts (Devaney & Henchion, 2018). By using different professionals, including researchers, advisors and producers, the current research aimed to provide realistic and practical prognoses based on a broad foundation, by incorporating different points of view. Finally, with the Delphi process, the current study not only aimed to obtain experts’ consensus on the future development of PAT in Swiss outdoor vegetable farming but also to identify promising measures to support adoption.

The focus of the current study was set on outdoor vegetable farming for three reasons. First, the agricultural area used for vegetable production has increased in Switzerland during the last decade (Zorn, 2020) and is very resource-intensive, for example in terms of pesticide and fertiliser use. Second, there is growing societal concern about the negative environmental impacts of agriculture, which is reflected, for example, in seven popular initiatives launched in Switzerland in 2016, all of them addressing agricultural or food-related topics (Huber & Finger, 2019). A current example is the so-called drinking water initiative. This popular initiative will directly affect vegetable farming by only giving direct payments to farms that preserve biodiversity and do not use pesticides (Huber & Finger, 2019; Schmidt et al., 2019). Farmers and policy makers are therefore urgently looking for ways to reduce the environmental impact of agriculture, with vegetable producers finding themselves at the forefront of technology adoption. Third, new technologies have the potential to reduce the negative environmental impacts of agricultural production. The results of this study identify promising technologies in this regard and show how measures can be taken to promote technology adoption in order to make agriculture more sustainable.

Focusing on outdoor vegetable farming in Switzerland, the present exploratory study followed three main objectives. First, it aimed to identify the drivers and barriers in technology adoption. The second aim was to obtain a prediction of the possible future development of the adoption of digital technologies based on the assessments of various experts. The third aim was to explore the possible political, regulatory and infrastructural measures that can assist technology adoption.

## Materials and methods

To investigate these research aims, a Delphi study was conducted. The Delphi method is an established tool for scientific forecasting, the main aim of which is to obtain high-quality responses from a selected panel of experts (Devaney & Henchion, 2018). Developed mainly by Dalkey and Helmer (1963), it aims to obtain a convergence of opinion in order to address future scenarios. Most Delphi studies share four main characteristics (Anderhofstadt & Spinler, 2019; Rowe & Wright, 2001; von der Gracht, 2012). First, the experts are anonymous, and their identity remains unknown to the expert panel, thus avoiding one or a few experts dominating the consensus process. This anonymity also helps avoid the bias caused by other group mechanisms, such as group pressure (Dalkey & Helmer, 1963). Second, the format of rounds offers experts the possibility to change or modify their statements. Third, experts are provided with controlled feedback, which summarises the results of the previous rounds, making it possible for them to reconsider their opinions (Hsu & Sandford, 2007). Fourth, the Delphi moderator provides feedback as a statistical group response, usually including measures of central tendency (e.g., mean, median).

### Procedure

The experts were contacted beforehand and informed about the study and its timeline. Their willingness to participate was also assessed, and when they agreed, they were added to the expert panel. Data were collected using the online survey tool Unipark (Questback GmbH, 2017). The data collection took place from October to December 2020 in two rounds. As in previous research (Kent & Saffer, 2014), the data collection took two weeks per round. In each round, the experts were asked to fill in the questionnaire within one week. Each participant received an individual expert code, which ensured that their data were treated anonymously and, at the same time, allowed us to monitor the response rate and remind non-responders. One week after sending out the survey, the non-responders were prompted to complete the questionnaire (Kent & Saffer, 2014). The following week was devoted to data analysis.

**Fig. 1:**
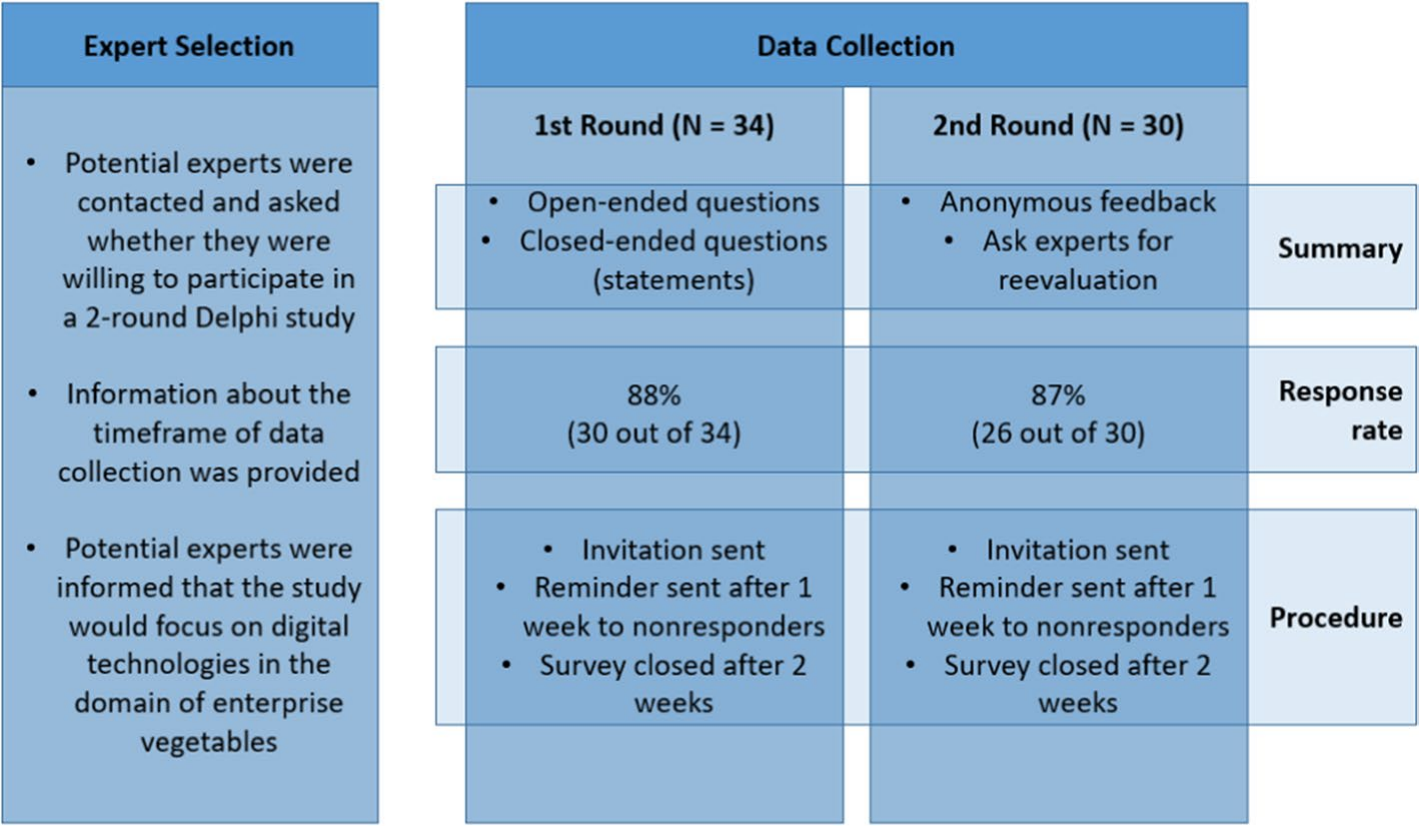
Study design for the 2-stage Delphi survey

In Round 2 of the Delphi study, the experts were informed about the results from Round 1 and asked for their feedback. Depending on the type of question, statistics depicting the results from Round 1 were provided, including mean values, number of mentions and figures depicting these results. Considering the limited time resources of the experts, the aim was to achieve minimum panel mortality by conducting two Delphi rounds only (Alon et al., 2019). The survey procedure is illustrated in Fig. 1.

### Expert selection

The selection of experts is a crucial step in all Delphi studies. While traditional survey methods usually aim to obtain representative samples, the aim of the Delphi study is to obtain high-quality responses from a selected panel of experts (Devaney & Henchion, 2018). It is important for the success of Delphi studies that experts have appropriate domain knowledge (Rowe & Wright, 2001). Also, the expert panel should consist of a heterogeneous group of experts, from 5 to 20 individuals, covering various geographic locations (Belton et al., 2019; Häder, 2014; Rowe & Wright, 2001).

Potential participants were selected across Switzerland based on their recognised knowledge of and familiarity with vegetable production and precision agriculture technologies. Additional individuals were then contacted based on snowball sampling from the approached experts. For the selection, a special focus was placed on professional and geographical diversity (Häder, 2014; Mauksch et al., 2020). In accordance with Busse et al. (2014), five expert groups were defined based on the experts’ professional work, as follows: farmers/contractors, input suppliers, intermediates, research and advisory (see Table 1). Following the recommendation of Häder (2014), the study aimed for a minimum of five experts per group to ensure sufficient group sizes. The selected individuals were contacted by email and asked if they were willing to participate in the study.

**Table 1:** Overview of the composition of the selected Delphi expert panel

Expert group		# invited	R1	R2	Total
1	Farmers/contractors	8	8	5	
2	Input suppliers	8	7	7	
3	Intermediates (media, associations, etc.)	6	5	4	
4	Research	5	4	4	
5	Advisory	7	6	6	
	Response rate		88%	87%	76%

*R1* Delphi Round 1, *R2* Delphi Round 2. The distinction between the expert groups is not, in all cases, clear-cut. Some of the experts might be assigned to several groups

In total, more than 100 experts were contacted in Switzerland, of which 45 individuals considered themselves suitable and willing to participate in the study. To ensure that the experts had sufficient knowledge in both the fields of vegetable farming and precision agriculture technology, the panel was reduced to 34 individuals.

### Data collection

#### Survey round 1

**Fig. 2:**
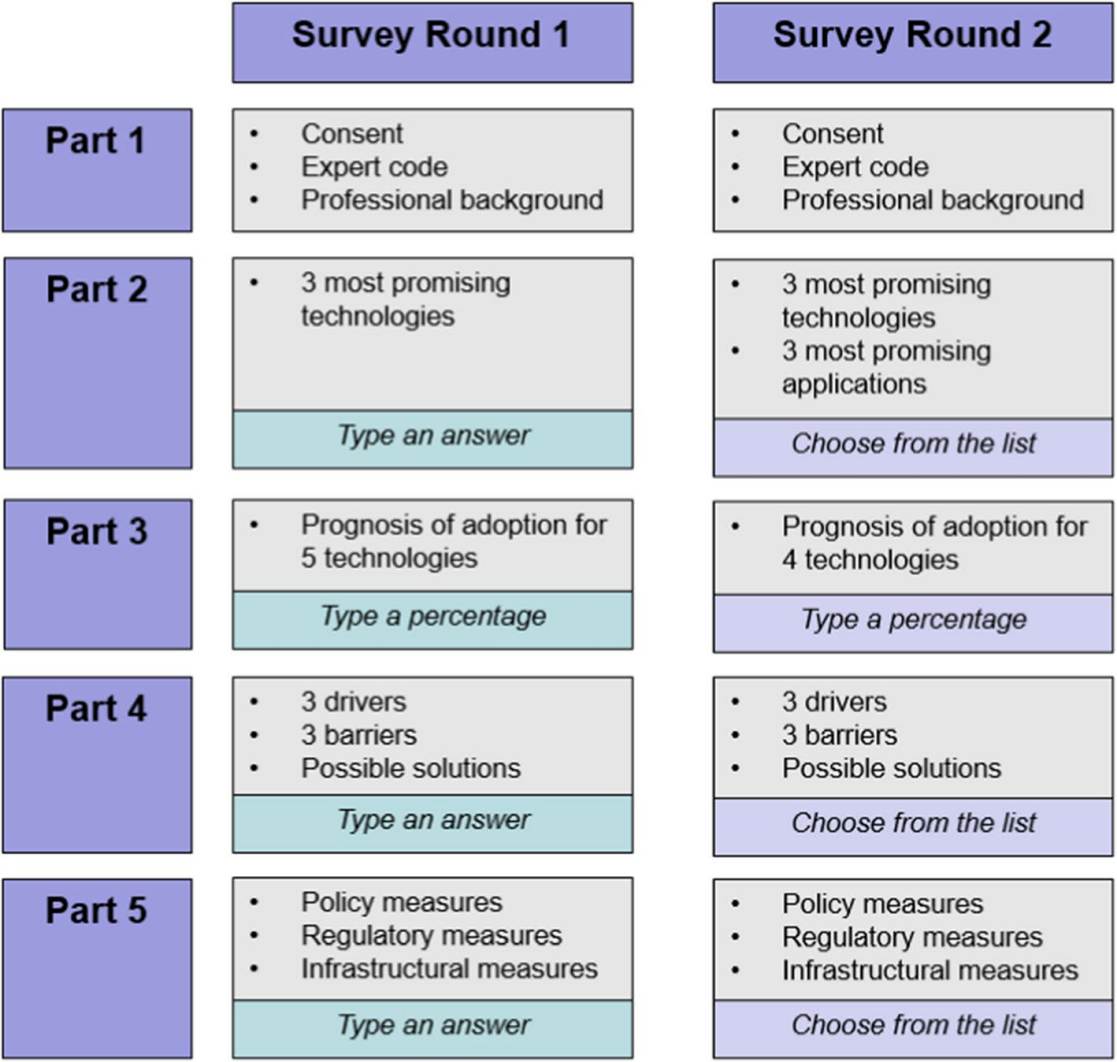
Overview on the survey structure for rounds 1 and 2

The survey consisted of five distinctive parts (see Fig. 2). In the first part, the experts were asked to provide their informed consent, their expert code and some information about their professional background, including their level of education. In the second part, they were asked to name at least three technologies they considered as the most promising for future developments in the domain of outdoor vegetable farming. A third part of the questionnaire informed the experts about the current level of adoption for five of the most frequently used technologies in Swiss outdoor vegetable production, using information from a representative survey on the status quo of mechanisation and digitisation in Swiss farming in 2018 (Groher et al., 2020). For each of the technologies, the experts were asked to estimate the short-term (the next 1 or 2 years), medium-term (the next 5 years) and long-term (the next 10 years) level of adoption. In a fourth part, the experts were asked to name at least three drivers and barriers they think are most important for the adoption of PAT. Subsequently, they were asked what measures could be taken to deal with the barriers they mentioned earlier. In a fifth and final part, they were prompted to think of possible measures in the domain of politics, regulatory environment and infrastructure that could help overcome the barriers to technology adoption.

As recommended in the literature (Häder, 2014), most questions were followed by a commentary field to give the participants the opportunity to further explain their choice or reasoning if they wished to do so. This also allowed the experts to name more than the minimum of three answers. Furthermore, the survey was kept as short as possible, making sure it did not exceed 30 min, in order to keep the experts’ motivations high (Okoli & Pawlowski, 2004).

Before sending out the questionnaire for data collection and, as recommended in the literature, a pre-test with four individuals not involved in this study was conducted (Marvin et al., 2020). These non-participating experts helped to ensure that the duration, clarity of the questions and completeness of the questionnaire were appropriate (Häder, 2014; Marvin et al., 2020).

#### Survey round 2

In the second Delphi round, the experts were given anonymous feedback on the results of Round 1, using mean values or numbers of mentions visualised in figures, and were asked again for their evaluation. For that purpose, the open-ended questions from Round 1 were transformed into closed-ended questions. The overall structure of the questionnaire remained the same as in the first round; however, two changes were made to the content. The first change concerns part two of the survey. In Round 1, the experts were asked to name promising technologies. Most of their answers specified both the technology and its application, since naming only one would be ambiguous. Consequently, it was decided to split the question into two parts, asking in Round 2 about both the technology and the application the experts considered as most promising. As a second change, in part three of the survey, where the experts estimated the future level of adoption for five technologies, spray drones were eliminated in Round 2 because the low numbers of adoption predicted by the experts in Round 1 indicated that they saw very little potential in that specific technology for vegetable farming.

### Data processing and statistical analysis

In Round 1, the experts provided some of their answers as text by using the text fields provided (see Fig. 2 for an overview). Given the qualitative nature of the resulting data, the responses were organised into groups. For instance, mentions including autonomous machines or various robots (e.g., hoeing robots) were summarised under the group *autonomous machines or robots*. After the data from Round 1 were collected, the answers were analysed and synthesised as feedback to be presented to the experts in Round 2. The first author did the analysis and grouped the qualitative data where appropriate and made sure the groups were phrased in a way that the experts were able to recognise their answers from Round 1. The second and third authors checked and validated the response groups before they were presented to the experts.

The results of Round 1 indicated that the 30 experts believed that spraying drones will play a minor part in the future of vegetable production. On average, they estimated the adoption rates of this technology in 10 years to be lower than 15%. Therefore, this question was excluded in Round 2. Analyses and visualisations were done using Microsoft Excel (2016) and IBM SPSS for Windows (version 24).

## Results and discussion

### Promising technologies and applications

**Fig. 3:**
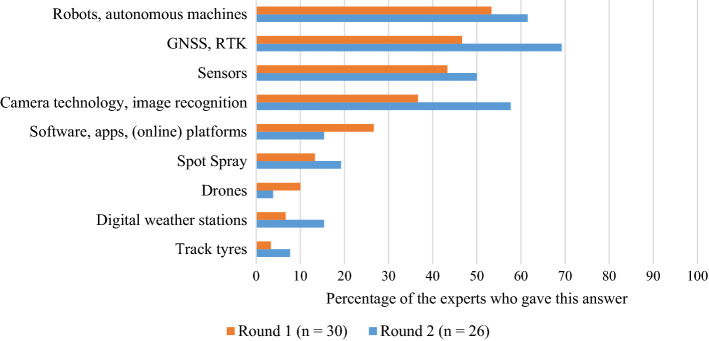
Most promising technologies according to the experts (data from Rounds 1 and 2). In Round 1, experts were asked to name at least three technologies; in Round 2, experts were allowed to choose no more than three from a given list of responses, as depicted

Based on the answers from Round 1 of the Delphi study, nine different groups of technologies were identified (see Fig. 3). In Round 2, the experts then selected the three groups they considered most important. The group of *GNSS*^1^*and RTK*^2^*technology* was the most promising in the experts’ view. This is not surprising, given that GNSS are commonly used in many countries, and most of the new machines farmers acquire are already equipped with this technology (Finger et al., 2019; Jochinke et al., 2007; Zhou et al., 2017).

The experts put *robots and autonomous machines* in second place (see Fig. 3). The popularity of this technology is supported by recent research from Germany, which found that 22.6% of the surveyed farmers were planning to invest in field crop robots within the next five years (Spykman et al., 2021). While *robots and autonomous machines* can bring significant benefits in terms of reductions in working hours or physical labour, their increasing use creates new challenges, such as legal concerns and health and safety issues. For instance, in the European Union, it remains unclear who is accountable for the damages caused by autonomous robots (Basu et al., 2018).

All the technologies mentioned by the experts have been on the market for some time now. Nevertheless, they encompass a range, from the more established technologies (e.g., driver assistance systems) to the newer and less established ones (e.g., drones). However, the mention of track tyres was rather surprising. Besides the question of whether this counts as a PAT, it is interesting to understand in what context the experts chose to mention it. One expert explained their choice as follows: “Track tyres are expensive and not very common in Switzerland. Since vegetable-growing soils are under a lot of strain and soil as a resource is scarce and in demand, I personally think that every effort must be made to preserve the Swiss vegetable-growing soils and take care of the soil structure”. This comment makes it clear that the health and quality of the soils in vegetable farms are of concern. Especially in farms where large areas of land are cultivated, heavy machines are used, which can cause soil compaction. Increasing the use of PAT can lead to a shift away from the heavy machines operated by farmers to the use of smaller, autonomous machines (King, 2017).

**Fig. 4:**
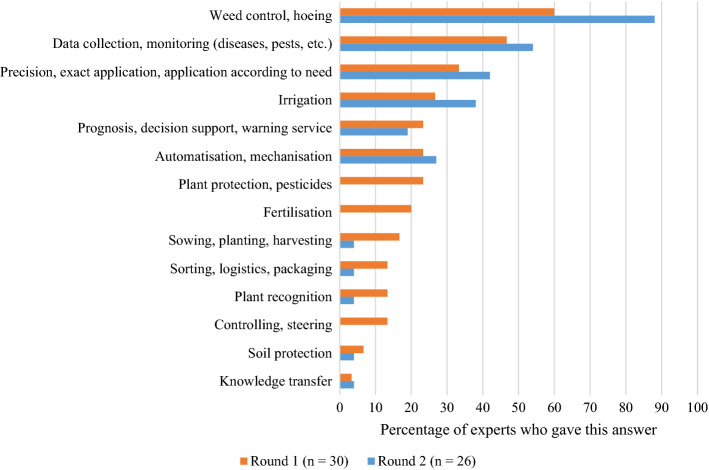
Most promising applications for new technologies according to the experts. In Round 1, experts were asked to name at least three; in Round 2, experts were allowed to choose no more than three from the responses collected in Round 1

In terms of promising applications, *weed control and hoeing* was a clear favourite, with 88% of the experts choosing it in Round 2 (see Fig. 4). Given the increasing societal and environmental pressure on agriculture in Switzerland and around the world, it seems that experts see significant potential in technologies concerning weed control and hoeing. These technologies can help lower the use of input in managing these tasks. Similarly, increased data collection and monitoring can help adjust crop farming practices in a way that input allocation is optimised. Therefore, it is not surprising that the second group of technology applications, *data collection and monitoring*, was selected by more than half of the expert panel.

### Future scenarios for adoption

**Fig. 5:**
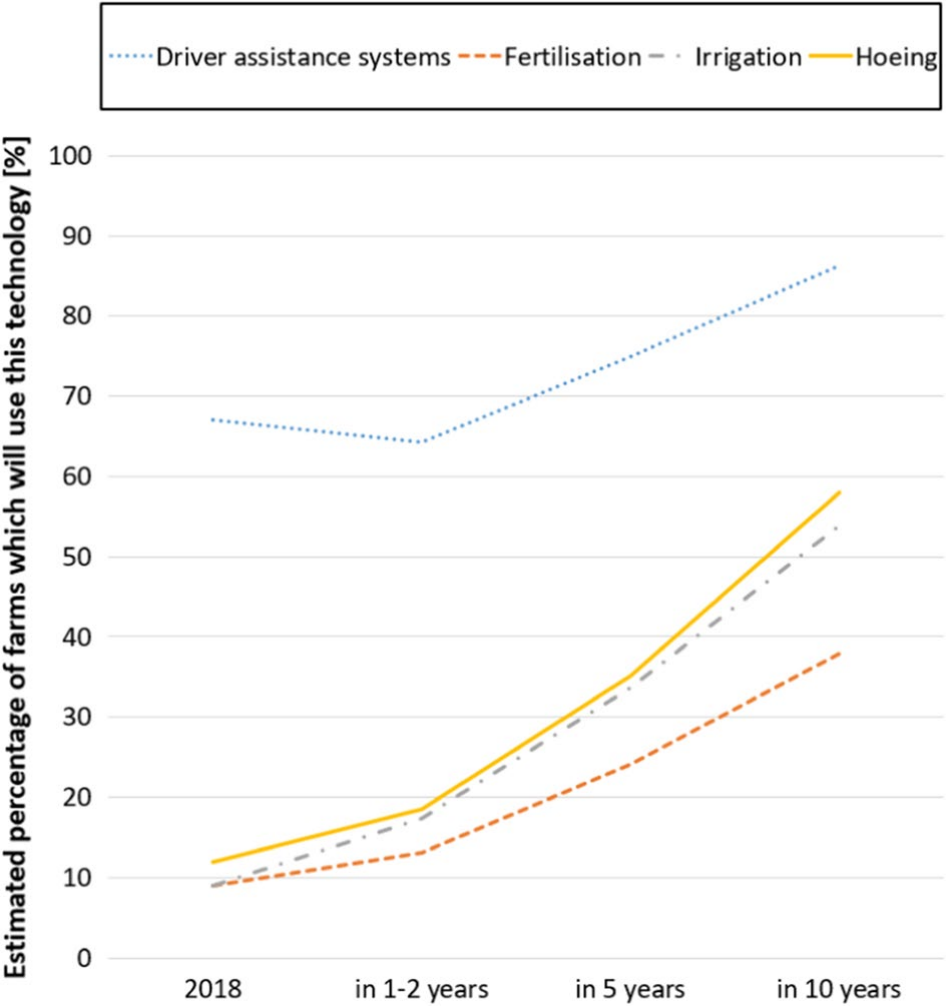
Expert prognoses for the level of adoption for driver assistance systems, sensors in fertilisation, sensors in irrigation and sensors in hoeing in the near, medium and far future in Round 2 (n = 26). Levels of adoption in 2018 were provided to the experts as a baseline

Figure 5 shows the experts’ predictions regarding the four technologies for which the highest adoption rates were reported in 2018 (Groher et al., 2020). The prognoses for the adoption rates of PAT are especially promising for irrigation and hoeing, possibly because these fields are under significant pressure due to current issues such as climate change and protection of the environment (for instance, through bans on pesticides). The expected adoption rates may be of interest to educators, researchers and technology marketers alike. Experts in the current study expect the adoption rates in the domains of fertilisation, irrigation and hoeing to almost double in the next one or two years. In the next 10 years, they expect them to grow by four times or more compared to the 2018 level (Groher et al., 2020). This expected increase will also significantly affect the demand for technology supply and training.

Surprisingly, the mean of the expected percentage of farms which will use driver assistance systems in one to two years is lower than the value experts were given as a baseline for the year 2018. However, there is no indication of a decrease in this use, and it is rather unlikely that farms, which already use the technology, would get rid of it. Therefore, it can be assumed that the experts must have missed or misinterpreted the information provided in Round 2, as this effect was absent in the results from Round 1.

### Drivers and barriers in the adoption of new technologies

**Fig. 6:**
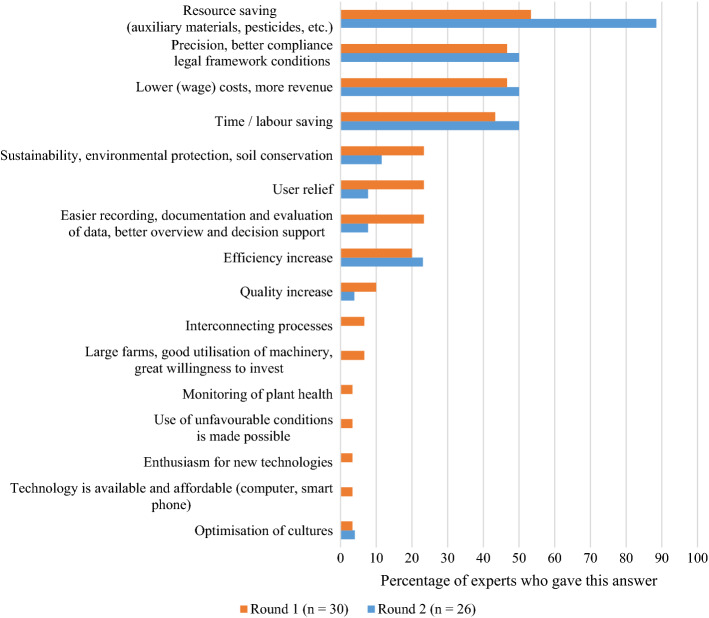
Drivers of adoption compiled from Rounds 1 and 2

When asked about the drivers of adoption, 88% of the experts in Round 2 chose *resource saving* as most important (see Fig. 6). Half of the experts mentioned each of the following: *better compliance with the legal requirements*, *lower costs or higher revenues* and the *saving of time or labour.* These results make it clear that economic aspects play a dominant role as the drivers of adoption. Promising technologies (e.g., hoeing robots) may reduce the input use, but the main drivers here seem to be the economic aspects, as well as societal and political pressure. In support of this interpretation, one of the experts commented: *“From an environmental perspective, producers are forced to produce more sustainably”.* Again, this highlights the pressure under which vegetable farms are currently operating.

From Fig. 6, it is interesting to see that the most important aspects remained the same for both rounds. However, in Round 2, a clear focus was on the saving of resources. The aspect of *time and labour savings* may be of greater importance than ever but only reached 50% of the mentions. There is significant contextual overlap with *lower (wage) costs, more revenue*, which may explain the number of mentions. Vegetable farmers often rely on the help of foreign paid labour. However, anti-immigration sentiments and the current COVID-19 pandemic make it more difficult to hire wage workers from abroad and thereby may increase the pressure to adopt PAT (Christiaensen et al., 2020).

**Fig. 7:**
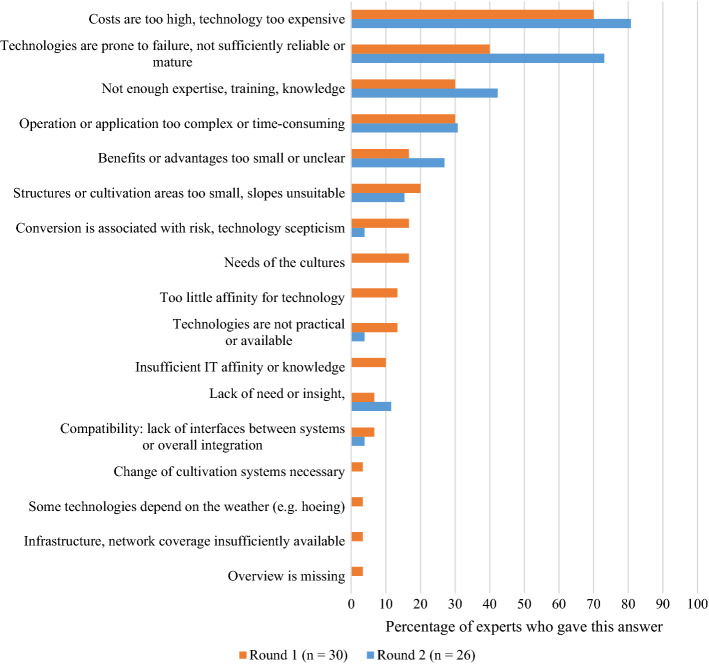
Barriers to adoption compiled from Rounds 1 and 2

When the experts were asked about the possible barriers to technology adoption, it emerged that the overall patterns were similar between the two rounds, with a more accentuated picture emerging in Round 2 (see Fig. 7). The *high costs* and the *level of technology development* were the most important barriers across both rounds. It is well documented in the literature that technology costs are a major barrier to the adoption of new technologies (Lawson et al., 2011; Reichardt & Jürgens, 2008). Similarly, farm size is a strong predictor for uptake, as larger farms tend to have more capital they can invest (Barnes et al., 2019). The mention of the *level of technology development* indicates that for some of the users, it may seem too early to adopt the technology. A survey conducted in Germany revealed that especially large farms were among the early adopters and that large amounts of time in the initial stages were required to operationalise the technology (Reichardt & Jürgens, 2008). However, this process could be accompanied and facilitated by advisory services (Lawson et al., 2011). In line with this, the experts mentioned the *lack of knowledge, expertise or training* as the third important barrier. Not only do farmers need a certain degree of knowledge in order to operate a technology, but also their seasonal workers need to be able to deal with these new challenges. Similarly, farmers need a certain degree of affinity for technology to be able to work with PAT.

To accelerate technology adoption, the better choice should be the easier choice. As long as too much time, money or other efforts are required to adopt PAT, adoption will be slow and farmers will continue to work with the technology they already know and own. One expert highlighted this situation as follows: *“As long as the use of pesticides is cheaper than, for example, hoeing AND there are no legal requirements for this, one sticks to what one knows and what is best executable with the educational level of the available seasonal workers”.* Here again, it can be concluded that a certain amount of societal and political pressure can assist adoption in terms of providing additional incentives.

**Fig. 8:**
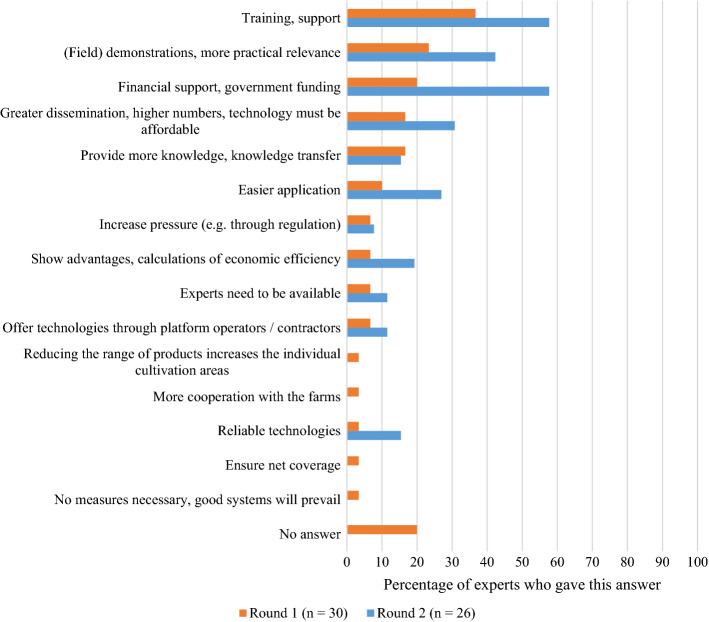
Possible solutions compiled from Rounds 1 and 2

In the next part of the survey, the experts were asked about possible solutions or measures that could assist adoption. In the first question, they were asked for solutions without being prompted in a specific direction. The high number of non-responders in Round 1 (20% of the experts), as well as the shift in the answers from Round 1 to Round 2, may be an indication that the experts had difficulty coming up with specific solutions as compared to identifying barriers and drivers. In Round 2, however, three answers emerged, with more than 40% of the experts’ mentions for each of them. The two most popular solutions, both mentioned by 58% of the experts, were *training* and *financial support* (see Fig. 8). These two aspects directly relate to the aforementioned barriers (costs and lack of knowledge). Specific training can help overcome possible knowledge gaps in technology use. For instance, a recent review identified a knowledge gap between measuring crop status and putting this information into action by making practical decisions in farm management (Balafoutis et al., 2020). The best technology is useless when the data it produces cannot be interpreted and put into practice.

Therefore, 42% of the experts mentioned *increasing the practical relevance*, which goes hand in hand with training and the demonstration of the economic benefits of these new technologies. Importantly, a recent study among teachers and students of the farm management course in Switzerland came to the same conclusion that practical relevance should be strengthened (Ammann et al., 2022). For instance, experimental fields and similar efforts can represent a huge potential for the promotion of new technologies. The increase of practical relevance also builds on communication efforts. As identified by previous research, communication can be of crucial importance in promoting PAT (Kutter et al., 2009), and positive communication among peers can help build confidence in the adoption of new technologies. Examples of this are several projects that have been launched in different countries. In Germany, the government initiated so-called experimental fields, which were built as pilot projects to test and refine digital technologies (Bundesministerium für Ernährung und Landwirtschaft BMEL, 2018). Further, a public-private partnership was launched in Switzerland in 2018 to create and demonstrate practical solutions for farmers (Swiss Future Farm, 2021).

**Fig. 9:**
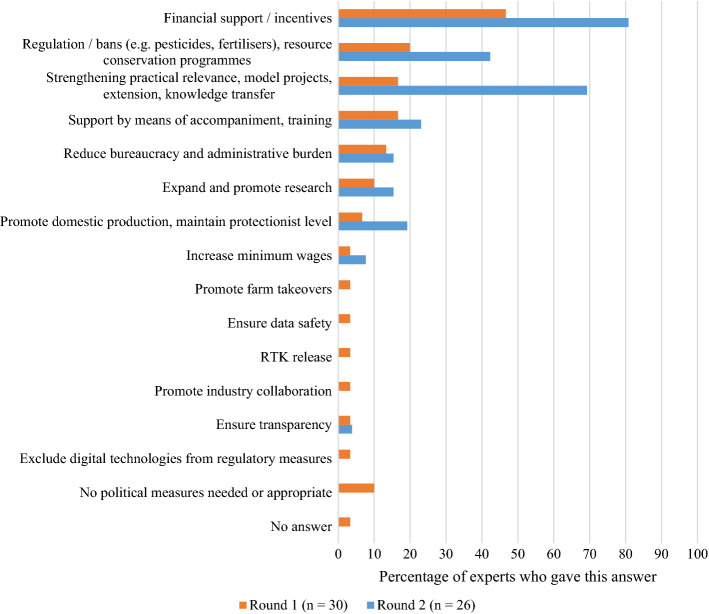
Political measures to support adoption of new technologies compiled from Rounds 1 and 2. For the reader’s convenience: it can be assumed that what is meant here by “RTK release” is “removal of RTK signal costs”

When specifically asked about the political measures that would support the adoption of new technologies, the experts again saw the biggest potential in *financial support* and *strengthening of the practical relevance*, such as through model projects (see Fig. 9). More than 60% of the experts in Round 2 mentioned both measures.

**Fig. 10:**
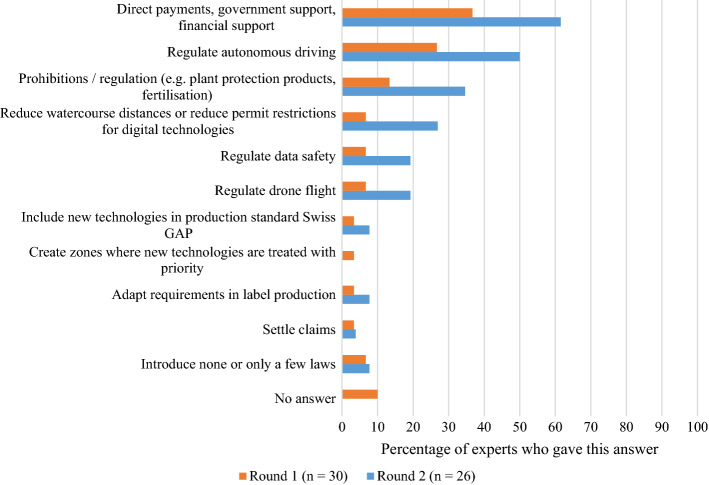
Regulatory measures compiled from Rounds 1 and 2

Similarly, when asked about regulatory measures that could assist the adoption of new technologies, *financial support* emerged as the most mentioned (see Fig. 10). One of the experts, however, expressed doubts or criticism about this and saw the financial responsibilities as lying primarily in the hands of the farmers: *“Promoting new technologies via direct payments usually does nothing. The payments are often far too low. The farms must be willing to invest in new technologies and take the risk”.*

It is interesting to note that *regulating data safety* received only 19% of the experts’ mentions in Round 2. Earlier in the questionnaire, when the barriers to technology adoption were investigated, data security was not mentioned at all. Similarly, in the context of possible political measures, other measures were rated as more important. Previous research conducted in Australia reported that, relative to other sectors, vegetable farmers had the least knowledge concerning their data and what types of agreements they had with their service providers (Wiseman et al., 2019). The current research further argues that in the domain of vegetable farming, data safety may be of less concern as compared to other agricultural branches, such as livestock farming. This observation is based on the fact that Swiss vegetable farmers are much less dependent on direct payments than their colleagues, meaning that data such as income statements and other business-related data must not be shared with the government. Still, when using new technologies, data is created and collected (Wolfert et al., 2017), and, in many countries, including Switzerland, a comprehensive regulatory framework around the collected data is missing. Furthermore, as many of these new technologies are still evolving, there are few security features in place (Rettore de Araujo Zanella et al., 2020). This, in turn, can be a hurdle for farmers in the adoption of these technologies (Wiseman et al., 2019) and, ultimately, makes it necessary to think about questions of data ownership, privacy and safety. While in the present study, data safety was a minor issue, it may be an important challenge vegetable farmers will face in the future.

**Fig. 11:**
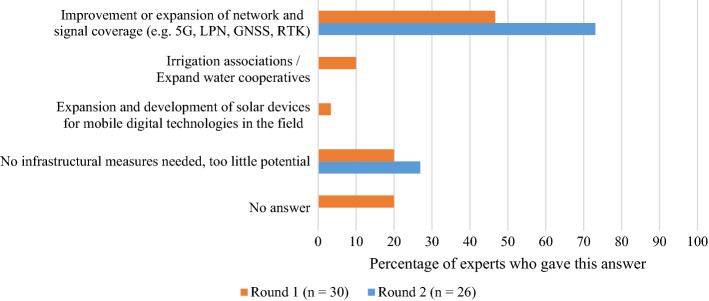
Infrastructural measures compiled from Rounds 1 and 2

Infrastructure is a basic prerequisite for many new technologies. Therefore, it is promising to see that this is the domain where experts saw the smallest potential for improvement. In total, 27% of the experts believed that no infrastructural measures were necessary, indicating that infrastructure is not the most urgent issue hindering the adoption of new technologies in Swiss vegetable production, which is mainly located in the valley regions. One of the experts summarised this view as follows: *“There are other more important problems than the infrastructure”.* The remaining 73% of the experts saw some potential in *improving the signal coverage* (see Fig. 11).

### Critical appraisal of the method

The range of the response rates of experts in the Delphi studies reported in the literature varies widely (Nowack et al., 2011). Some studies obtained response rates of below 40% (Keller & von der Gracht, 2014; Moldrup & Morgall, 2001; von Briel, 2018), while others reported response rates of around 51% (Kluge et al., 2020) or 70% (Mateos-Ronco & Server Izquierdo, 2011). Possible reasons for the high response rates of more than 75% in the present study are that the experts were contacted beforehand to indicate their willingness to participate and that they were informed about when the data collection would take place and how much time they would need to invest if they chose to participate.

A major limitation of the present study is that the participants were free to mention any technology that came to mind, but, in most cases, they did not further specify it. Given the wide range of technologies available, there is ample room for interpretation. Although the survey method did not allow us to follow up on answers that were not entirely clear, future research could use interviews to address this issue and thus overcome this specific limitation.

In all Delphi studies, the selection of experts is a crucial step. Given that no compensation was offered for study participation except for a short summary of the results, the present sample is subject to motivation bias. Of all the experts contacted, only around half replied that they were willing to participate in the study. However, the willingness of those experts who did agree to participate in exchange for a small report on the study results can be seen as an indication of a strong interest in the topic. Another difficulty in the interaction with the experts was that the method of relying on surveys made it difficult to put some of their answers into context. First, only a few of the experts added comments in the text boxes provided and second, there was some room for interpretation or a small degree of uncertainty in many of the comments.

A final issue to mention here regarding the Delphi method concerns its major strength and weakness. The survey format provided experts with a safe space where they could communicate their opinions in an anonymous way. However, most of the experts did not add any reasoning as to why they gave the answers they did. This made it hard, in some cases, to understand what exactly the experts meant by their answers or what their motivations were.

## Summary and conclusions

A two-stage Delphi study was conducted to predict the future development of technology adoption in outdoor vegetable production in Switzerland, to identify the drivers and barriers and to find suitable measures to support technology adoption. The results of this exploratory study indicate that economic factors are the crucial drivers and barriers in technology adoption. Furthermore, increasing the practical relevance emerged as a promising measure to assist this adoption. In that regard, this research is in line with previous findings, but it adds important insights which can help tailor policy and training measures aiming to increase the adoption of digital technologies. In particular, the experts identified a pronounced demand for financial support to overcome the cost barriers. Specific training, accompanied by advisory support, can help build more practical relevance and support farmers in technology adoption.

Synthesising the experts’ feedback on the most promising technologies and applications as well as their benefits reveals a scenario that encompasses the three domains of sustainability: the social, economic and environmental aspects. Robots and autonomous machines serve as the means to decrease wage costs (economic sustainability) and reduce physical strain by assuming tasks that are physically demanding (social sustainability). Among other tasks, GNSS/RTK technology is most commonly associated with navigation aids, such as driver assistance (social and environmental sustainability). Sensors, for instance, allow for the monitoring of plant or soil parameters and data collection, so that farmers do not have to go to their fields to take measurements (social and economic sustainability), and also allow for precise resource application (environmental and economic sustainability). Finally, camera technology and image recognition can facilitate weed control and monitoring of plant health without the farmers having to walk their fields (social and economic sustainability), as well as contribute to the precise application of auxiliary materials (environmental and economic sustainability).

The current level of adoption of PAT in vegetable farming indicates that considerable potential exists for growth in this area, and the experts predict steady increases within the next 10 years. The experts in the present study identified several measures that can help the adoption process. On the individual level, skills and IT knowledge are a crucial prerequisite in the ability to handle digital technologies. Policy makers can and should make sure that basic IT skills are obtained within the country’s compulsory schooling. In agricultural training, PAT should undoubtedly be part of the curriculum. Advisory services and farms that already use PAT can offer demonstrations to promote these technologies and help build confidence in their use. On a farm level, technology costs are an important barrier that can be overcome by financial support or through regulations (i.e., limits or prohibition of certain substances), which can serve as incentives to switch to new technologies. To unfold their potential, all these measures need to be built on a solid foundation based on agricultural education and accompanied by advisory services providing value-free support to farmers. Keeping this in mind can help improve efforts in training and policy measures aimed at supporting technology adoption. Undoubtedly, changes in climate and the regulatory framework directed at preserving natural resources will further increase the pressure on agriculture. PAT can play a key role in mastering these future challenges.

## Data Availability

The datasets generated and analysed during the current study are available from the corresponding author on request.

## Footnotes

1 Global navigation satellite systems.

2 Real-time kinematic.

## Code availability

The SPSS syntax for the respective analysis is available from the corresponding author on request.
